# Diffusion tensor tractography of the corticobulbar tract in a dysphagic patient with progressive supranuclear palsy: A case report

**DOI:** 10.1097/MD.0000000000032898

**Published:** 2023-02-10

**Authors:** In Jun Han, Hyeok Gyu Kwon, Woong-Woo Lee, Ra Gyoung Yoon, Hyoseon Choi, Hyun Jung Kim

**Affiliations:** a Department of Rehabilitation Medicine, Nowon Eulji Medical Center, Eulji University School of Medicine, Seoul, Republic of Korea; b Department of Physical Therapy, College of Health Science, Eulji University, Gyeonggi, Republic of Korea; c Department of Neurology, Nowon Eulji Medical Center, Eulji University School of Medicine, Seoul, Republic of Korea; d Department of Radiology, Nowon Eulji Medical Center, Eulji University School of Medicine, Seoul, Republic of Korea.

**Keywords:** corticobulbar tract, diffusion tensor tractography, dysphagia, progressive supranuclear palsy

## Abstract

This paper reports the changes over time in the corticobulbar tract (CBT) analyzed using diffusion tensor tractography (DTT) in a dysphagic patient with progressive supranuclear palsy (PSP).

**Patient concerns:**

A 53-year-old man initially presented with dysarthria, gait disturbance, and bradykinesia, and approximately 1-year later, downward gaze paralysis appeared. Initially, there was no dysphagia; however, approximately 2 years after visiting the hospital, symptoms of dysphagia, including difficulty swallowing pills, aspiration, and oral movement impairments appeared. The symptoms gradually progressed, and finally, mouth opening was severely damaged to the extent that it was difficult to orally feed.

**Interventions:**

We performed diffusion tensor imaging 3 times; at 3-month, 20-month, and 41-month from onset.

**Outcomes:**

On 3-month DTT, the left CBT was well reconstructed, whereas the right CBT showed partial tearing. In the 20-month DTT, both CBTs became thinner compared to the 3-month DTT. On 41-month DTT, both CBTs became much thinner than after 3-month and 20-month DTT.

**Lessons:**

We observed the degree of CBT injury over time in a dysphagic patient with PSP. These results suggest that the analysis of CBT using DTT is helpful in predicting the degree of dysphagia and prognosis in patients with PSP.

## 1. Introduction

Progressive supranuclear palsy (PSP) is an atypical parkinsonism characterized by early postural instability and decreased vertical eye movements. It is known that various symptoms such as rigidity and bradykinesia may appear in PSP, and bulbar symptoms such as dysphagia appear in the early stages compared to Parkinson disease.^[1,2]^ Aspiration pneumonia is the most common cause of death in PSP^[3]^; therefore, evaluation of the course and prognosis of dysphagia in PSP is very important. In addition, the prognostic evaluation of dysphagia in PSP is helpful in determining treatment strategies such as whether to apply aggressive treatment at an early stage and whether invasive attempts such as gastrostomy are necessary.

Swallowing is a complex process involving several cranial nerves, motor nerve nuclei, peripheral sensory nerves, and muscles. Two major systems, the cortical and subcortical systems, are involved in the brain, and the corticobulbar tract (CBT) connects the 2 systems.^[4]^ The swallowing process can be evaluated through a videofluoroscopic swallowing study (VFSS).

Diffusion tensor imaging (DTI) is a type of magnetic resonance imaging technique that quantifies the diffusion of water molecules in vivo to determine the physiological characteristics and abnormalities of tissues.^[5]^ Diffusion tensor tractography (DTT) is a 3-dimensionally reconstructed neural tract based on diffusion tensor images, which has the advantage of quantification.^[6,7]^ The DTT technique has been used in studies comparing the degree of dysphagia and CBT injury in patients with cerebral infarction and cerebral hemorrhage.^[8,9]^ However, to the best of our knowledge, no studies on the correlation between the degree of dysphagia and the prognosis of dysphagia in patients with PSP, and no study on the correlation between the degree of dysphagia and the degree of CBT injury has been reported.

Therefore, in this case report, by analyzing the changes over time of CBT using DTT and VFSS results of a case of PSP, we investigated the clinical usefulness of the DTT technique in predicting the prognosis of dysphagia.

## 2. Case presentation

**Figure 1.**
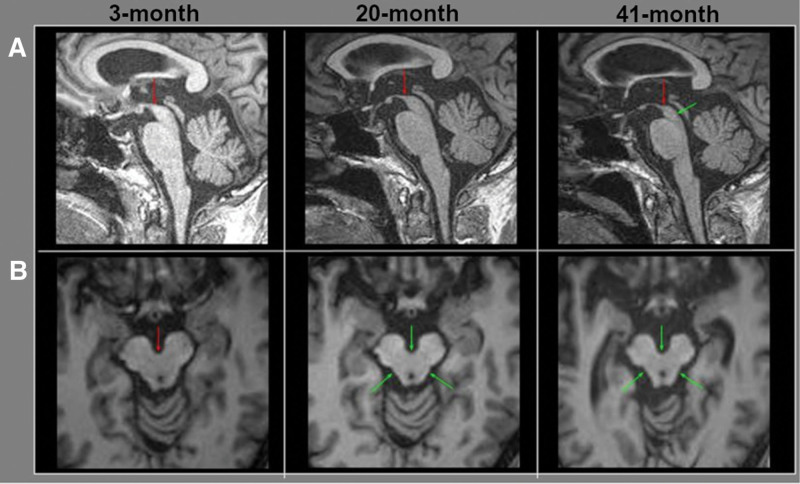
MR images of midbrain in a progressive supranuclear palsy patient. (A) Sagittal T1-weighted image. (B) Axial T1-weighted image. On the 3-month MRI, there were minimal and subtle changes in the midbrain (red arrows). Subsequently, flattening of the superior surface of the midbrain was apparent (red arrow), and the AP diameter at the level of interpedencular groove was slightly decreased (green arrows) on 20-month MRI. Progressive flattening (red arrow) and reduction of the volume (green arrows) was remarkable on 41-month MRI. MRI = magnetic resonance imaging. AP = anteroposterior.

A 53-year-old man visited the hospital complaining of dysarthria, gait abnormality, and bradykinesia that occurred 2 months before the outpatient visit. No symptom of dysphagia was observed at that time, and serial studies such as brain magnetic resonance imaging (MRI) and FP-CIT PET CT scans were performed 3 months after the onset of the primary chief complaint. On the 1^st^ MRI performed 3 months after symptom onset, subtle flattening of the superior surface of the midbrain was suspected on sagittal T1WI (Fig. 1A). In FP-CIT PET CT scan, unlike typical Parkinson disease, in which DAT binding is reduced in the posterior putamen in the early stage and in the caudate nucleus in the later stage, both areas were severely reduced, suggesting atypical parkinsonism, especially PSP. Twenty months after the onset, the patient underwent an MRI scan due to severe downward gaze paralysis. Flattening of the superior surface of the midbrain was apparent on the 2^nd^ MRI performed 20 months after symptom onset, which was consistent with the hummingbird sign (Fig. 1A). In addition, the anteroposterior diameter at the level of the interpedencular groove was slightly decreased and showed a slightly concave lateral margin of the tegmentum of the midbrain (Fig. 1B). However, even when he was hospitalized for additional diagnostic tests, he did not complain of dysphagia. We performed VFSS to identify latent dysphagia, and observed delayed swallowing reflex. The Dysphagia Outcome and Severity Scale score at this time was 6, which indicates mild functional impairment that allows a normal diet. Subsequently, symptoms of difficulty swallowing pills appeared for the first time, and symptoms of aspiration and food retention in the mouth appeared in turn. Repetitive VFSS revealed aspiration on a liquid diet, followed by oral phase dysfunction, such as impairment in tongue movement, mastication, and bolus formation. After 41 months of onset, we performed the 3^rd^ MRI to check the progression state, and the flattening and concave configuration of the midbrain progressed (Fig. 1). At this time, VFSS showed aggravation of oral phase dysfunction, and Dysphagia Outcome and Severity Scale was 2, which indicates severe dysphagia in which oral feeding is almost impossible. In the last VFSS, 45 months after onset, it was difficult to perform the test because of severe oral phase dysfunction, including mouth opening. In the pharyngeal phase, the silent aspiration was observed with liquid trial. This study was approved by our institutional review board and the requirement for written consent was waived (EMCS 2022-11-006).

## 3. Diffusion tensor imaging

We performed DTI 3 times (at 3-month, 20-month, and 41-month after onset). We acquired the DTI data using a 3T MR scanner Skyra (Siemens Healthineers, Erlangen, Germany) with a standard 8-channel phase array head coil. For each of the 15 non-collinear and non-coplanar diffusion-sensitizing gradients, we acquired approximately 70 contiguous slices parallel to the anterior commissure-posterior commissure line. The imaging parameters of DTI were as follows: 2 b values with b = 0 and b = 1,000s/mm^2^; 30 diffusion directions; voxel size 2 × 2 × 2mm; matrix = 120 × 120; field of view = 240 × 240mm^2^; repetition time = 8000ms; echo time = 78ms; parallel imaging reduction factor 2; echo-planar imaging factor = 51; b = 1000s/mm^2^; number of excitations, 1; and a slice thickness of 2mm. Affine multi-scale 2-dimensional registration at the Oxford Centre for Functional Magnetic Resonance Imaging of Brain (FMRIB) software library (FSL; www.fmrib.ox.ac.uk/fsl) was applied to correct the image distortion and head motion effect due to eddy currents.^[10]^ We performed fiber tracking using a probabilistic tractography method based on a multi-fiber model, and applied in the current study utilizing tractography routines implemented in FMRIB Diffusion (5000 streamline samples, 0.5 mm step lengths, curvature thresholds = 0.2).^[10–12]^ For reconstruction of the CBT, the seed region of interest was placed on the lower portion of the precentral gyrus on the axial image with the level of the top of the lateral ventricle.^[13,14]^ The target region of interest was positioned in the CBT area (between the transverse pontine fibers and the middle cerebellar peduncle) on the axial image with the level of the mid-pons.^[13,14]^

**Figure 2.**
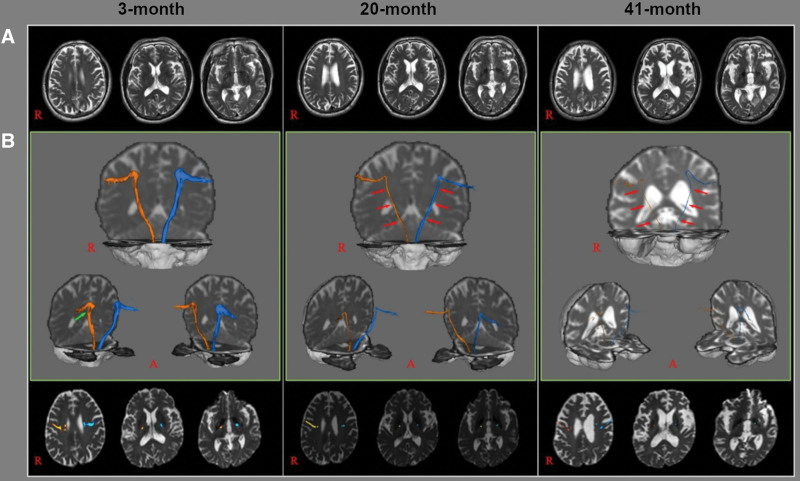
MR images of brain and diffusion tensor tractography (DTT) images for the corticobulbar tract (CBT). (A) Axial T2-weighted image of brain (B) On 3-month DTT, well reconstructed CBT is shown in the left hemisphere; in contrast, the right CBT showed partial tearing (green arrow). Both CBTs in 20-month DTT became thinner (red arrows) compared to 3-month DTT and on 41-month DTT, both CBTs were much thinner (red arrows) than 20-month DTT. CBT = corticobulbar tract, DTT = diffusion tensor tractography.

On 3-month DTT, a well-reconstructed CBT was shown in the left hemisphere; in contrast, the right CBT showed partial tearing. However, in the 20-month DTT, both CBTs became thinner than in the 3-month DTT. Furthermore, on 41-month DTT, both CBTs became much thinner than after 3-month and 20-month DTT (Fig. 2).

## 4. Discussion

In this study, we investigated the change in CBT using DTT as dysphagia progressed in a patient with PSP. In the patient, dysphagia appeared first as pharyngeal phase symptoms, such as difficulty swallowing pills and aspiration, followed by oral phase symptoms, such as biting in the mouth. Repeated swallowing tests over time showed a delayed swallowing reflex in the first test, followed by aspiration of liquids, and finally, oral phase dysfunction. In addition, deterioration of the oral phase was more prominent than deterioration of the pharyngeal phase, and even mouth opening was damaged on the last VFSS, making it difficult to perform the test.

Regarding DTT results, partial tearing of the right CBT was observed in the initial 3-month DTT, and both CBTs were thinner than before in the 20-month DTT. While the abnormal findings on conventional MRI began to appear at the 20-month (Fig. 1), the DTT findings were markedly different in 20-month (Fig. 2). Diffusion tensor tractography can more sensitively reflect the degree of progression of PSP. In addition, although both CBTs became remarkably thin in the 20-month DTT, the patient did not complain of dysphagia, and even in the VFSS performed 3 months from 2^nd^ DTT, only minor finding, that is delayed swallowing reflex was observed. Thus, it can be considered that clinical dysphagia symptoms appear when damage to both CBTs progresses beyond a certain level. In addition, the period between each DTT was not significantly different at 17 and 21 months, but there was little difference in clinical symptoms between 3-months and 20-month, while the difference between 20-month and 41-month was very noticeable. From this, it can be considered that dysphagia symptoms will progress rapidly when the damage of CBT on DTT exceeds a certain level in patients with PSP, which can be helpful in predicting the prognosis of patients with PSP. Therefore, further studies based on the results conducted in a larger number of patients are needed.

Recently, studies using DTI in PSP patients have attempted to identify characteristically damaged areas in PSP, which are differentiated from Parkinson disease or healthy subject.^[15–17]^ In addition, there are studies on the relationship between dysphagia and CBT in various diseases such as cerebral infarction and hemorrhage through DTT.^[8,9]^ However, the significance of this study is that, to the best of our knowledge, this is the first case study to demonstrate the relationship between dysphagia and CBT changes in patient with PSP.

In previous studies, dysphagia in PSP first appeared with oral phase problems,^[18]^ but in this patient, symptoms related to the pharyngeal phase appeared initially, followed by symptoms related to the oral phase. This can be explained by previous studies showing that both hemispheres play different roles in the swallowing process. According to previous studies, the left and right hemispheres are responsible for the oral and pharyngeal phases, respectively.^[19–21]^ In this patient DTT, the right CBT was damaged first, and both sides were damaged in the second DTT, but the right side was damaged more severely. Therefore, it can be assumed that symptoms of the pharyngeal phase (responsible for the right hemisphere) were prominent. However, further studies with larger numbers of patients are needed.

A previous study confirmed the location of CBT in the internal capsule through DTT, located in the middle and third portions of the posterior limb, not the previously known genu in the internal capsule.^[22]^ In the DTT image of this study, CBT was located in the middle and third portions of the posterior limb, and it can be used as evidence to support the previous study.

This study had several limitations. First, since this study was a single case report, there are limitations in generalizing the conclusions of this study. Second, it is possible that the DTT results were affected by false positive and false negative effects caused by crossing fibers or partial volume effects^.[23]^ Therefore, readers should exercise caution in the interpretation of this study.

## 5. Conclusion

This study is the first to investigate the relationship between dysphagia in PSP and CBT damage using diffusion tensor tractography. We found that dysphagia in a PSP patient began to appear and progressed rapidly when the damage to the CBT exceeded a certain level. Therefore, it is possible to predict the degree of dysphagia in patients with PSP through diffusion tensor tractography, which is helpful in predicting the prognosis of patients with PSP. However, further studies with larger numbers of patients are required.

## Author contributions

**Conceptualization:** Hyun Jung Kim.

**Investigation:** In Jun Han.

**Formal analysis:** Hyeok Gyu Kwon.

**Funding acquisition:** Hyeok Gyu Kwon.

**Resources:** Woong-Woo Lee, Ra Gyoung Yoon, Hyoseon Choi.

**Supervision:** Hyun Jung Kim.

**Visualization:** Hyeok Gyu Kwon.

**Writing – original draft:** In Jun Han.

**Writing – review & editing:** Hyeok Gyu Kwon, Woong-Woo Lee, Ra Gyoung Yoon, Hyoseon Choi, Hyun Jung Kim.
